# Small bowel lipoma and intussusception: a case report

**DOI:** 10.1093/jscr/rjae327

**Published:** 2024-05-28

**Authors:** Fahreyar Alam, Stewart Chikukuza, Omar Okkeh, Filip Tsvetkov, Zulfiqar Hanif, John Lawrence, Richard Payne

**Affiliations:** Department of General Surgery, Great Western Hospital NHS Trust, Swindon SN36BB, UK; Department of General Surgery, Great Western Hospital NHS Trust, Swindon SN36BB, UK; Department of General Surgery, Great Western Hospital NHS Trust, Swindon SN36BB, UK; Department of General Surgery, Great Western Hospital NHS Trust, Swindon SN36BB, UK; Department of General Surgery, Great Western Hospital NHS Trust, Swindon SN36BB, UK; Department of Pathology, Great Western Hospital NHS Trust, Swindon SN36BB, UK; Department of General Surgery, Great Western Hospital NHS Trust, Swindon SN36BB, UK

**Keywords:** small bowel lipoma, intestinal intussusception, small bowel obstruction

## Abstract

Intussusception is defined as the telescoping of bowel into itself. Intussusception is the leading cause of bowel obstruction in children, but it is rare in adults [1, 2]. It has a pathological intramural or extramural lead point. In adults, it accounts for 1%–5% of cases of bowel obstruction [3, 4]. Unlike presentation in the paediatric population of cramping abdominal pain, bloody mucus, and palpable mass in right iliac fossa, presentation in adults can be more varied and non-specific [1, 4]. Hence, diagnosis of small bowel intussusception (SBI) can be challenging, requiring a higher degree of clinical suspicion [5]. While cases of paediatric intestinal intussusception are often primary, most adult cases are secondary to structural lesions [5]. This case is of a 57-year-old lady who presented with SBI secondary to a small bowel lipoma (SBL), reflecting the importance of considering SBL as a differential in the causes of SBI.

## Introduction

Intussusception was first mentioned by Barbette of Amsterdam in 1674 [6]. It was described in detail in 1789 by John Hunter, who described it as a rare form of bowel obstruction in adults [7].

Benign causes in adults include Crohn’s disease, endometriosis, hamartoma, infections, Kaposi sarcoma, lipoma, Meckel diverticulum, neurofibroma, polyps (inflammatory, adenomatous), stromal tumours, and tuberculosis. Malignant causes include adenocarcinoma, carcinoid tumours, leiomyosarcoma, lymphoma, malignant gastrointestinal stromal tumours, metastatic carcinoma, and neuroendocrine tumours [8].

This case looks at SBL as the lead point for intussusception-causing small bowel obstruction (SBO), requiring an emergency laparotomy.

Lipomas of the gastrointestinal tract are uncommon and have a reported frequency of 0.15%–4.4%, with the majority developing in the large intesine [2, 9]. They are the second most common benign tumour of the small bowel after leiomyoma [10]. They are slow growing tumours and common between the fifth and seventh decade of life with a slight female preponderance [10]. Small bowel is the second most common location for lipomas of the gut. About 20–25% of lipomas occur in the ileum. They are usually pedunculated and submucosal. The submucosal type is most common, followed by the intermuscular and serosal types. Although mainly asymptomatic and usually an incidental finding, symptoms are related to size, location, and mobility due to the pseudo pedicle [10]. Larger lesions >2 cm invariably lead to intussusception. Symptoms include intussusception causing bowel obstruction, GI bleeding from ulceration, constipation, and diarrhoea [2, 10].

Diagnosis of intussusception from lipomas is challenging due to the varied presentation in patients. Despite advances in medical imaging, only 32%–50% of cases are diagnosed preoperatively [5]. Computerized Tomography of Abdomen and Pelvis (CTAP) with contrast is the gold standard for diagnosis [4, 8]. The finding of a homogeneous mass with Hounsfield units between −80 and −120 is nearly pathognomonic [10]. A CT scan is particularly useful for the detection of larger lipomas (>2 cm) [10].

Due to an underlying pathological lead point, the treatment of choice in adult population for SBO secondary to SBI from a lipoma is laparotomy plus small bowel resection, unlike non-operative reduction with barium or air in the paediatric population, where in most cases there is an absence of a pathological lead point [2, 3].

The length of small bowel involved in an intussusception should always be considered, as it can result in short bowel syndrome.

## Case report

We present a case of a 57-year-old female patient who presented to the emergency department (ED) with a week’s history of abdominal pain, distention, not passing stool or flatus, and feculent vomiting. She denied any fever, melena, or diarrhoea. She had no significant past medical or surgical history and no previous abdominal surgery. She was haemodynamically stable and apyrexial. A general physical examination revealed class III obesity with no pallor, jaundice, or lymphadenopathy. Abdominal examination revealed a distended, soft, mildly tender abdomen with no palpable masses or visceromegaly. Hernial orifices were clear, and the rectal examination was unremarkable.

Her routine blood tests revealed an Hb of 172 g/L. Her C-reactive protein was elevated at 239 mg/L, and she had an acute kidney injury (AKI), urea of 16.8 mmol/L, and normal creatinine.

**Figure 1:**
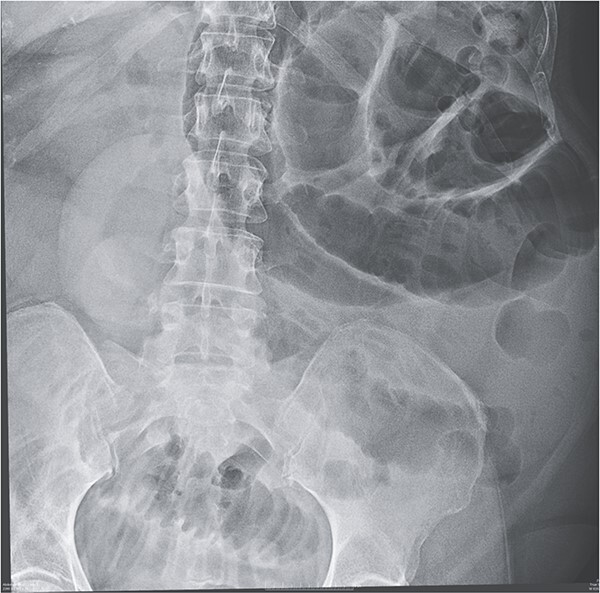
X-ray showing multiple dilated small bowel loops.

**Figure 2:**
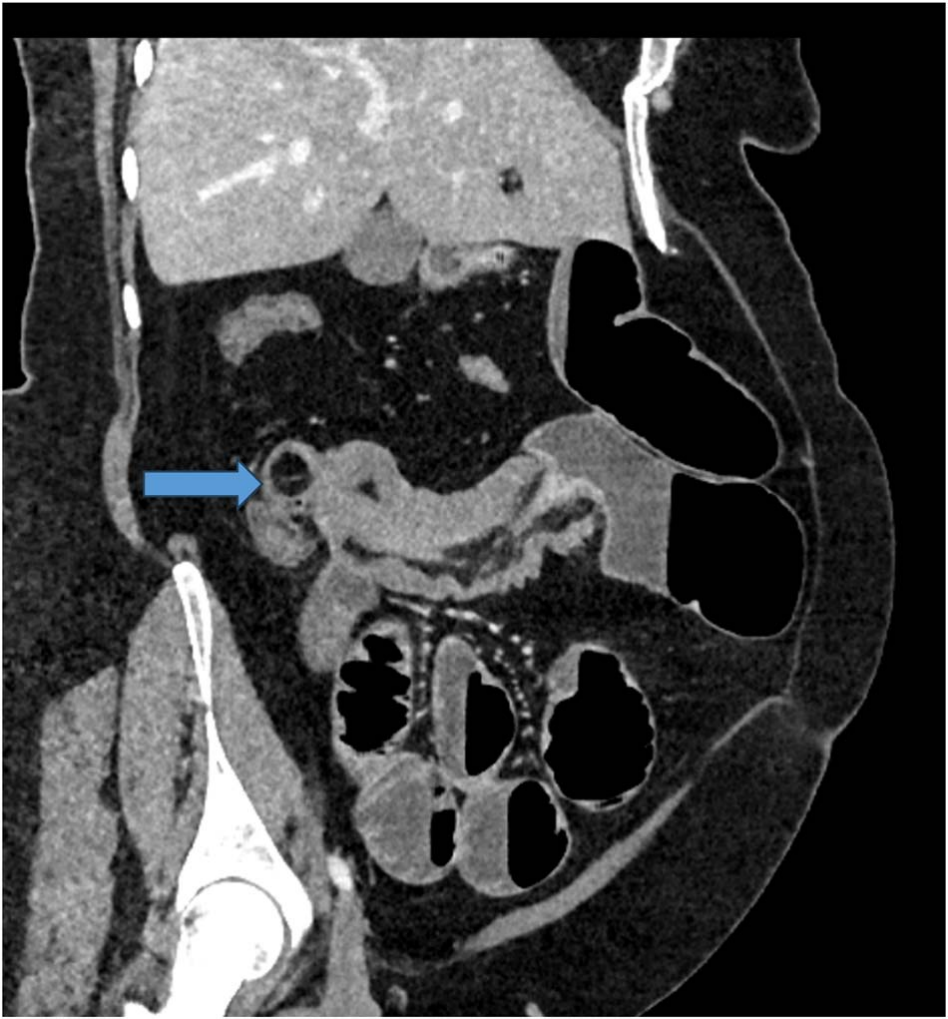
CT scan of abdomen: oblique coronal view of abdomen showing intussusception with low attenuation lesion distally C/W a lipoma (arrow).

**Figure 3:**
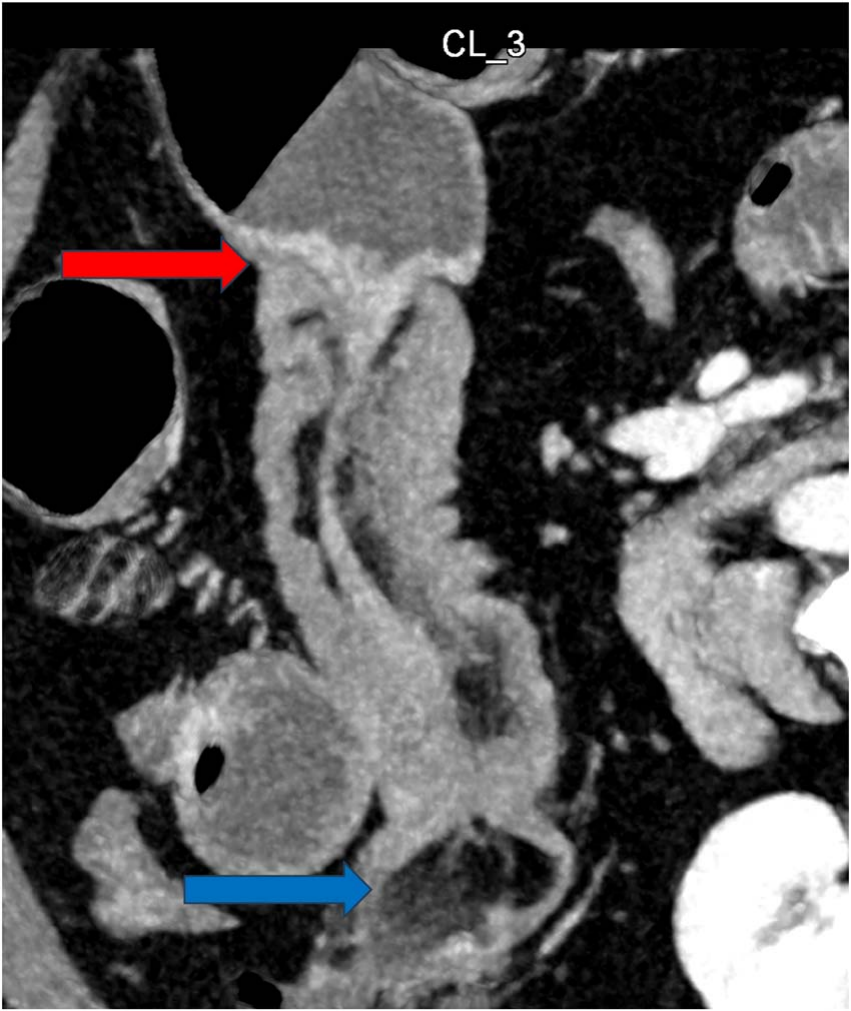
CT Abdomen: MPR reconstruction of the plane of intussusception showing a distal lipoma and proximal start of intussusception.

Given her abdominal symptoms and signs, she was subjected to an abdominal X-ray (Fig. 1), which showed multiple distended loops of the small bowel, and a chest X-ray, which showed left basal atelectasis. A working diagnosis of SBO was made. She was put nil by mouth. A nasogastric tube and a urinary catheter were inserted. After initial resuscitation, she was subjected to CTAP with contrast. The findings of the CTAP were in keeping with acute distal SBO secondary to an ileo-ileal intussusception. No obvious mucosal mass was stated in the initial report (Figs 2 and 3).

**Figure 4:**
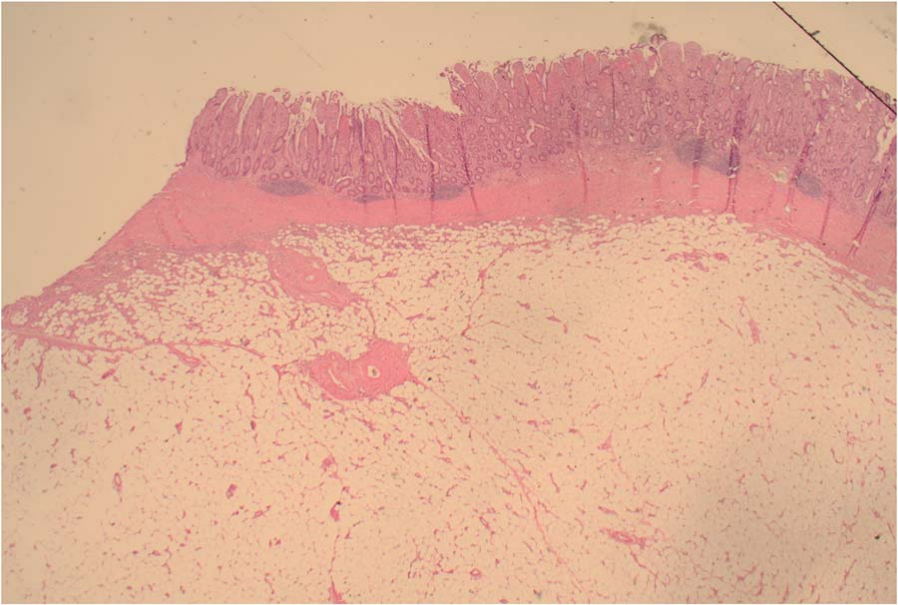
12.5× magnification showing the intact surface small bowel mucosa with the underlying lipoma.

**Figure 5:**
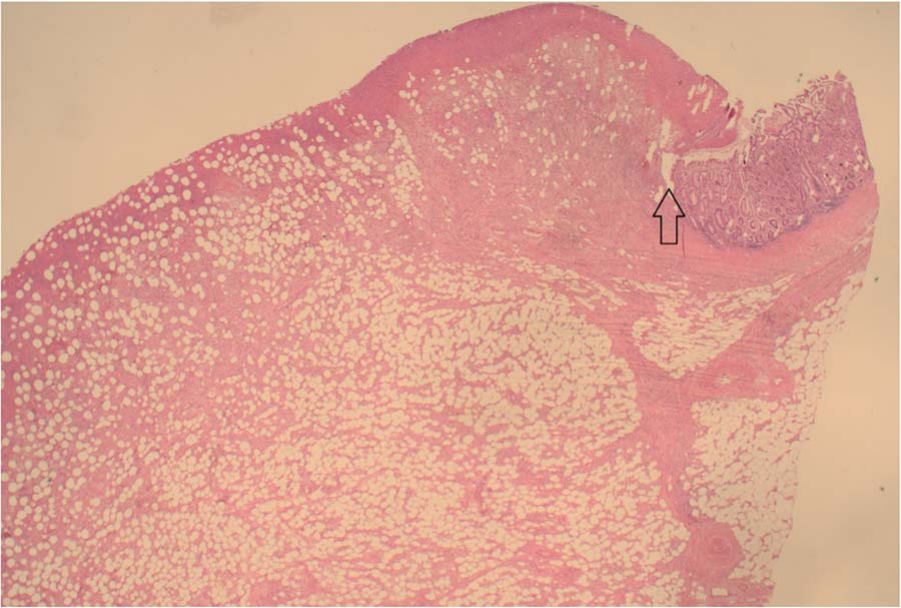
12.5× magnification showing the junction between the ulcerated leading point of the intussusception on the left and the preserved mucosa on the right of the arrow.

**Figure 6:**
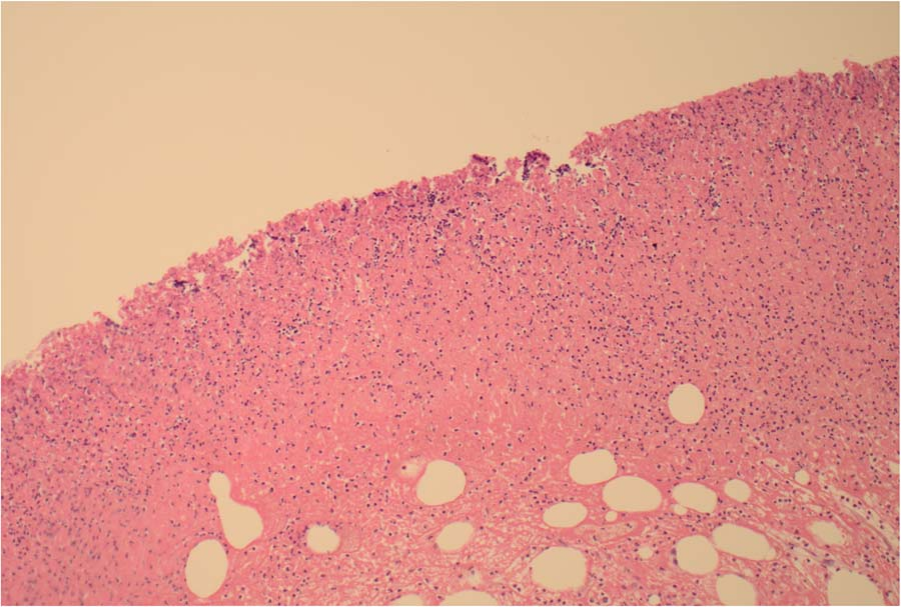
100× magnification showing the ulcerated surface of the lipoma which is covered by fibrin and neutrophils. The clear cystic spaces below this are the residual fat cells.

**Figure 7:**
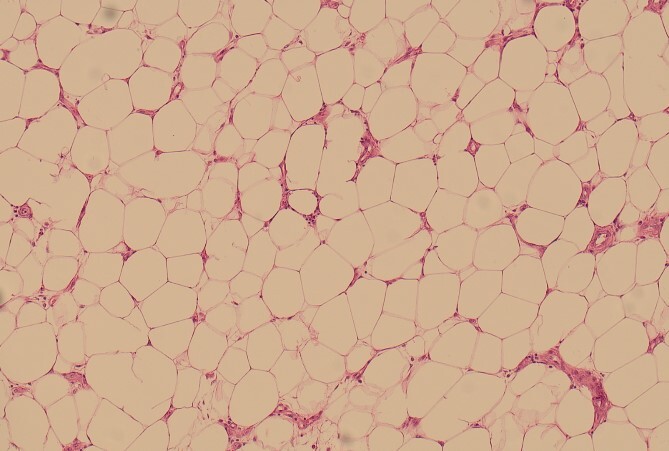
100× magnification of the lipoma illustrating the adipocytes which show cytoplasmic distention by optically clear fat.

The patient was taken to the theatre for an emergency laparotomy. Intraoperative findings were of high-grade SBO, secondary to terminal ileal intussusception. The involved small bowel was viable with no perforation. The intussusception was reduced. Nidus was an intraluminal polypoid lesion. A small bowel resection and a primary, double-layered hand-sewn anastomosis were performed. A specimen was sent for histology. Histology confirmed an ulcerated benign submucosal lipoma (45 × 30 × 22 mm) as the cause (Figs 4–7).

The patient had an uneventful postoperative recovery with no residual symptoms and was discharged home on the seventh postoperative day.

## Conclusion

While cases of paediatric intestinal intussusception are often primary or idiopathic, most adult cases are secondary to structural lesions. Diagnosis of SBI in adults can be challenging due to the varied presentation, and clinicians need to have a high index of suspicion. Although lipomas of the gastrointestinal tract are uncommon, they should always be considered as a possible aetiology in cases of SBO secondary to intussusception.

This case report is a good example of an adult case of SBI secondary to a structural lesion (small bowel lipoma).

CTAP with contrast is the gold standard for diagnosis, but in this case, it didn’t show lipoma as a mucosal cause, further suggesting having small bowel lipoma as a differential for such cases.

In order to minimize the length of the resected small bowel, we strongly advocate intraoperative reduction of the intussuscepted segment prior to definitive surgical management.
